# Anti-inflammatory and chondroprotective effects of the S-adenosylhomocysteine hydrolase inhibitor 3-Deazaneplanocin A, in human articular chondrocytes

**DOI:** 10.1038/s41598-017-06913-6

**Published:** 2017-07-25

**Authors:** Juliette Aury-Landas, Céline Bazille, Lyess Allas, Sara Bouhout, Christophe Chesneau, Sylvain Leclercq, Karim Boumédiene, Catherine Baugé

**Affiliations:** 1 Normandie University, UNICAEN, EA7451 BioConnecT Caen, France; 2 CHU de Caen, Service d’Anatomie Pathologique, Caen, France; 3 Normandie University, UNICAEN, CNRS UMR 6139 LMNO Caen, France; 4 Clinique Saint-Martin, Service de Chirurgie Orthopédique, Caen, France

## Abstract

3-Deazaneplanocin A (DZNep) is an inhibitor of S-Adenosyl-L-Homocysteine Hydrolase (SAHH) known to inhibit EZH2, a histone methylase upregulated during osteoarthritis. In this study, we assessed its effects in human articular chondrocytes. Anti-inflammatory effects were assessed by Nitric Oxide (NO), Prostaglandin E2 (PGE2) and Metalloprotease (MMP) release in IL-1β-stimulated chondrocytes. MAPK and NFκB activation was analyzed by western blotting. Differentially expressed genes (DEG) regulated by DZNep were identified by whole-transcriptome microarray. DZNep inhibited SAHH activity and was not toxic. It counteracted NO, PGE2 and MMP release, and reduced MAPK activation induced by IL-1β. By whole-transcriptome analysis, we identified that DNZep counteracts the effect of IL-1β on the expression of 81 protein-coding genes, including *CITED2*, an MMP inhibitor. These genes are organized in a protein-protein network centred on EGR1, which is known to functionally interact with EZH2. Gene ontologies enrichment analysis confirmed that DZNep counteracts IL-1β-induced expression of genes involved in cartilage matrix breakdown (*MMPs* and *ADAMTS*). In addition, DZNep up-regulated cartilage specific genes, such as *COL2A1* and *SOX9*, suggesting a chondroprotective effect of DZNep. DZNep exhibits anti-inflammatory effects, and regulates genes implicated in chondroprotective response in human articular chondrocytes, suggesting that inhibitors of S-adenosylmethionine-dependent methyltransferases could be effective treatments for OA.

## Introduction

Cytokines, particularly Interleukin-1 beta (IL-1β), play a major role in the development and progression of osteoarthritis (OA). IL-1β is highly present in the synovial fluid of OA patients and contributes to the degradative process of cartilage in causing overproduction of Prostaglandin E2 (PGE2), Reactive Oxygen Species (ROS), Nitric Oxide (NO) and metalloproteinases (MMPs). These MMPs, produced by chondrocytes, cause severe joint damages. In particular, MMP-1 and MMP-13 induce the digestion of type II Collagen, the principal collagenous material in hyaline cartilage, while MMP-3 is involved in the breakdown of extracellular matrix by degrading proteoglycans.

IL-1β induces its pro-inflammatory effect through the activation of protein kinase cascades involving the MAP Kinase (MAPK) and/or NFκB signalling pathways. The MAPK pathway consists of ERK, p38 and JNK. These MAPK subtypes are constitutively expressed in articular chondrocytes and transiently activated by numerous stimuli, including IL-1β. Several studies suggest that MAPK are also able to modulate the IL-1β–induced NFκB pathway. NFκB normally exists as an inactive cytoplasmic complex bound to inhibitory proteins of the IκB family. Its induction requires the activation of IκB/kinase complex that phosphorylates IκB molecules, triggering their degradation. Release of IκB results in the nuclear translocation of NFκB and its binding to specific DNA sites, inducing the transcription of target genes, such as *MMPs*. Interestingly, MAPK as well as NFκB pathway have been shown to be activated in OA cartilage and can play a key role in the cartilage destruction seen in OA^1, 2^.

IL-1β is one of the most important cytokines involved in the pathogenesis of OA, disturbing the catabolism and anabolism processes and causing considerable pain and disability^3, 4^. Therefore, the use of pharmacological treatments to inhibit IL-1β effects presents a useful strategy to treat OA^5^. In addition, IL-1β treatment of chondrocytes is an easy way to mimic inflammatory part of osteoarthritis *in vitro*, permitting to assess the interest of anti-arthritic molecules. Such studies have highlighted the anti-inflammatory effects of tested drugs by measuring the repression of expression of inflammatory markers, such as MMPs, PGE2 or NO levels, as well as the activation of MAPK and NFκB pathways induced by IL-1β^6^.

Recently, 3-Deazaneplanocin A (DZNep), a carbocyclic adenosine analog derived from the naturally occurring antibiotic neplanocin A, has been reported to have anti-inflammatory effects. For instance, in the context of allogenic graft, this molecule reduces the inflammatory infiltration in liver, intestine and skin of recipients^7^. In this study, both interferon-γ (INF-γ) and tumor necrosis factor-α (TNF-α) were down-regulated by DZNep indicating an ameliorated inflammatory environment in recipients. Besides, in breast cancer cells, microarray analysis showed that DZNep regulates numerous genes involved in inflammation supporting that this analog may regulate a wide range of genes involved in inflammation network^8^. Also, DZNep induces significant decreased inflammatory phenotypic cytokines, including TNF-*α*, IL-6, IL-12, IL-1β and MCP-1, in RAW264.7 cells and in murine polarized macrophages obtained from mice treated with lipopolysaccharide plus IFN-*γ* ^9^. Together, these data suggest that DZNep may be an innovative molecule to counteract inflammation process during OA.

DZNep is a potent inhibitor of S-Adenosyl-L-Homocysteine Hydrolase (SAHH), a component of the methionine cycle^10^. SAHH catalyzes the reversible hydrolysis of S-Adenosyl-L-Homocysteine (SAH) to adenosine and homocysteine, which is converted to methionine. When this enzyme is inhibited, SAH accumulates in cells, leading to inhibition of the histone methyltransferase (HMT) activity and the subsequent histone methylation inhibition. In particular, DZNep has been reported to specifically inhibit the histone methylase EZH2 leading to a decrease of the methylation of the histone H3 on the lysine 27 (H3K27)^11^. Interestingly, EZH2 has been recently described as a regulator of chondrocyte proliferation and hypertrophy^12, 13^. In addition, EZH2 level was found significantly increased in chondrocytes of OA patients compared to normal humans, suggesting that this methylase may be involved in OA development^13^.

In this study, we assessed *in vitro* the effects of DZNep in human articular chondrocytes. We showed that this inhibitor of S-adenosyl-L-homocysteine hydrolase and EZH2 histone lysine-N-methyltransferase has anti-inflammatory, anti-catabolic and chondroprotective effects since it reduces IL-1β-induced MAPK activation, NO and PGE2 production and MMP expression while it upregulates Sox9 and COL2A1. We suggest also a putative role of *CITED2* and *EGR1* in this response.

## Results

### DZNep is not toxic for chondrocytes and inhibits SAHH

**Figure 1:**
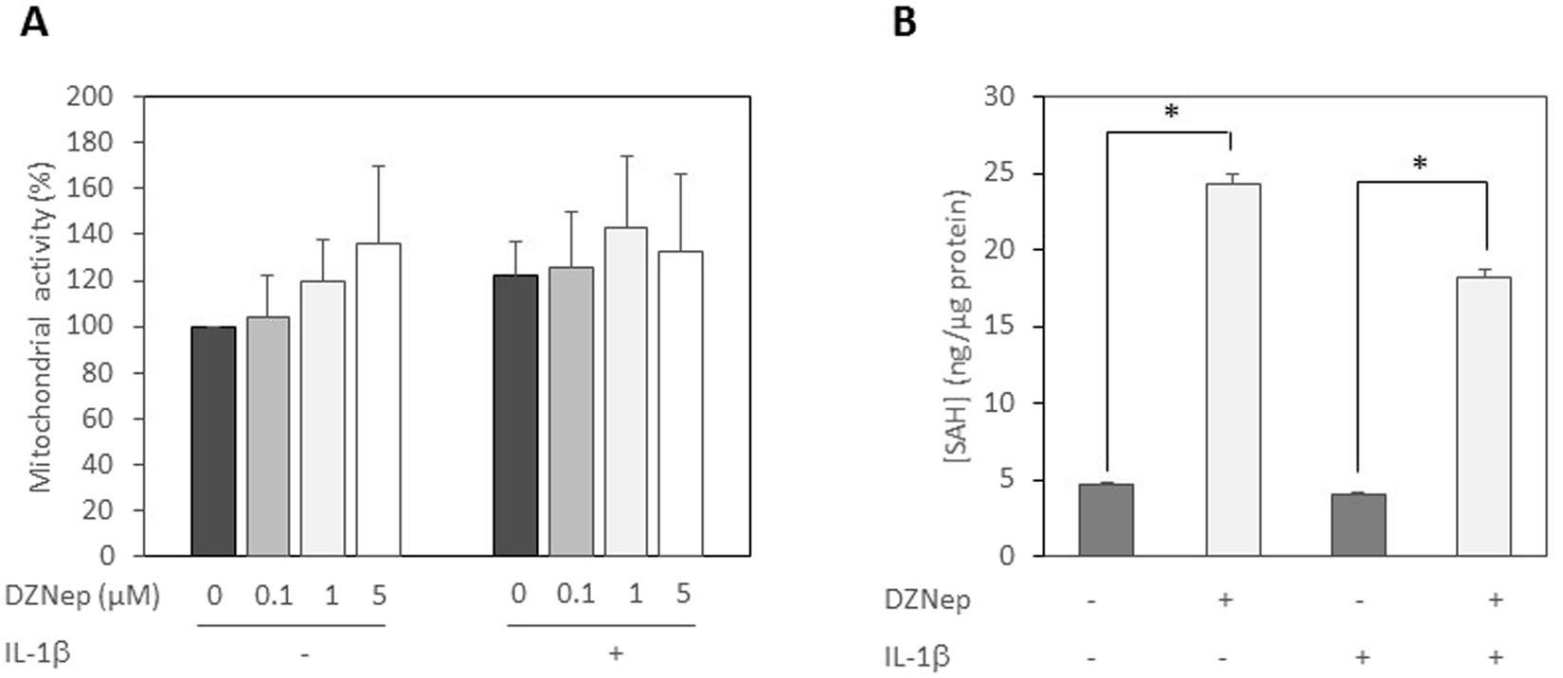
DZNep, a SAHH inhibitor, was not toxic in chondrocytes. (**A**) Primary chondrocytes were treated with DZNep (0 µM, 0.1 µM, 1 µM or 5 µM) in the presence or absence of IL-1β (1 ng/mL) for 72 h. After treatment, cell viability was assessed by WST-1 assay. Data are expressed as means ± SEM. (**B**) Primary chondrocytes were treated or not with DZNep (1 µM) in the presence of IL-1β (1 ng/mL) for 72 h. After treatment, proteins were extracted, and SAH levels were determined in cells by ELISA assay. Data are expressed as means ± SEM. *p-value ≤ 0.05.

DZNep was initially used for its ability to induce apoptosis in tumoral cells, while it has been described to be safe for normal cells. We first assessed the toxicity of DZNep on human chondrocytes. Primary chondrocytes were treated 72 h by increasing doses of DZNep in the presence or absence of IL-1β. Cells treated with DZNep showed no sign of cytotoxic effects or any negative effects on cell viability under light microscopy. Measurement of the metabolic activity by WST-1 assay confirmed that DZNep (0.1 to 5 μM) was not toxic for human chondrocytes (Fig. 1A).

Next, inhibitory activity of DZNep against SAH hydrolase in human chondrocytes was measured by the elevation in SAH concentration in proteins extracted from whole cells^10^. As expected, DZNep treatment induced SAH intracellular accumulation in both untreated and IL-1β-stimulated chondrocytes (Fig. 1B).

### DZNep reduces NO and PGE2 production induced by IL-1β

**Figure 2:**
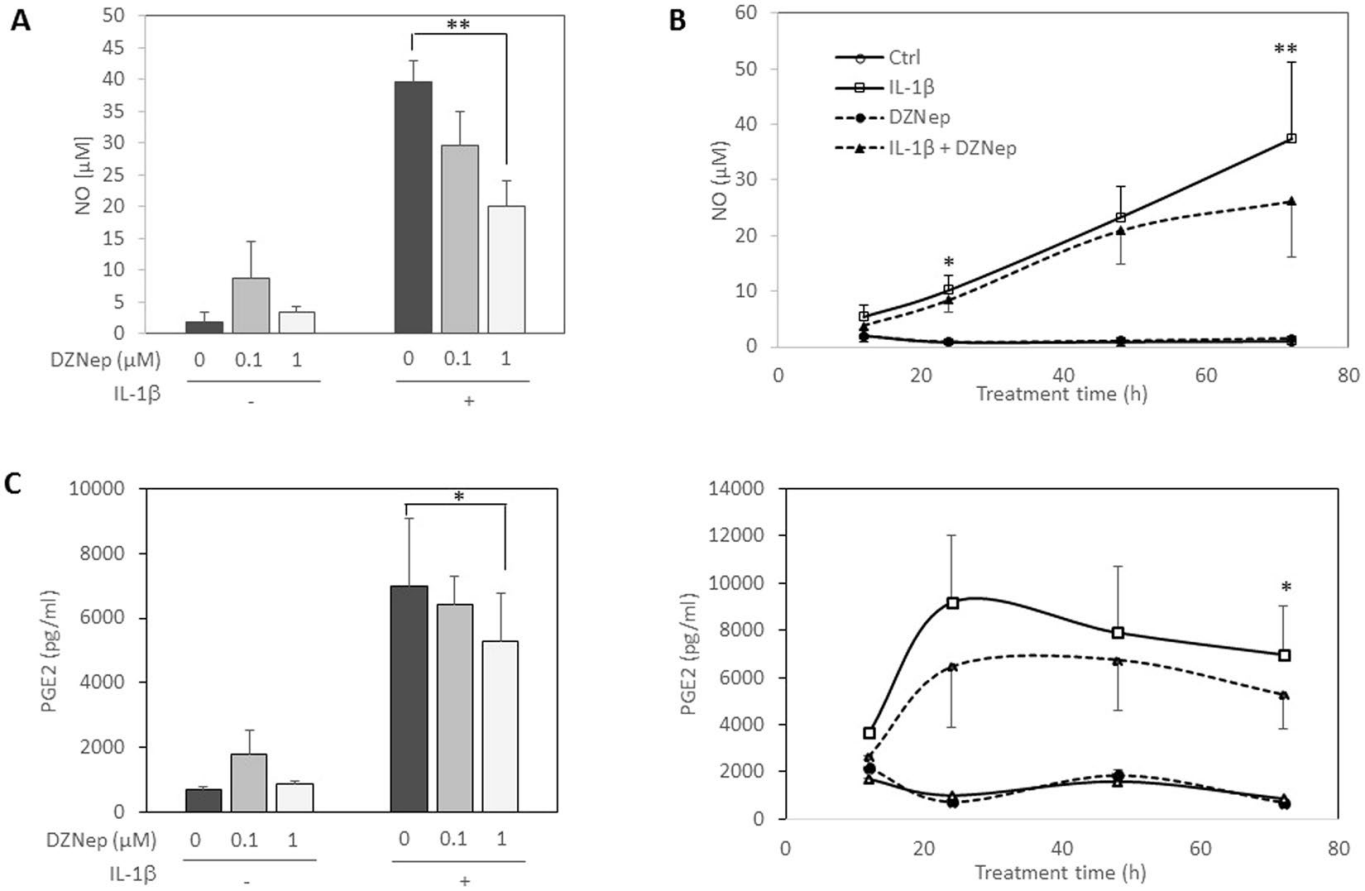
DZNep reduced NO and PGE2 production induced by IL-1β. (**A**) Primary chondrocytes were treated or not with DZNep (0 µM, 0.1 µM or 1 µM) in the presence or absence of IL-1β (1 ng/mL) for 72 h. (**B**) Primary chondrocytes were treated or not with DZNep (1 µM) in the presence or absence of IL-1β (1 ng/mL) for 12 h, 24 h, 48 h or 72 h. After treatment, NO production was measured in culture medium by Griess reaction assay. (**C**) Cells were treated or not with DZNep (1 µM) in the presence or absence of IL-1β (1 ng/mL) for 72 h. After treatment, PGE2 was quantified in culture medium by ELISA assay. Data are expressed as means ± SEM. Statistical significance between “IL-1β” and “IL-1β + DZNep” conditions are represented. *p-value ≤ 0.05; **p-value ≤ 0.01.

Then, we investigated the effect of DZNep on the production of NO induced by IL-1β. A 72 h-treatment with DZNep prevented IL-1β-stimulation of NO release in medium in a dose-dependent manner (Fig. 2A). The dose of 1 µM of DZNep was chosen for the following experiments as this dose reduced significantly the production of NO induced by IL-1β. Time course experiments (from 12 h to 72 h) confirmed that DZNep counteracted the induction of NO by IL-1β (Fig. 2B).

Additionally, DZNep (1 µM) significantly reduces the PGE2 release in IL-1-treated chondrocytes (Fig. 2C).

### DZNep counteracts MMPs release induced by IL-1β

**Figure 3:**
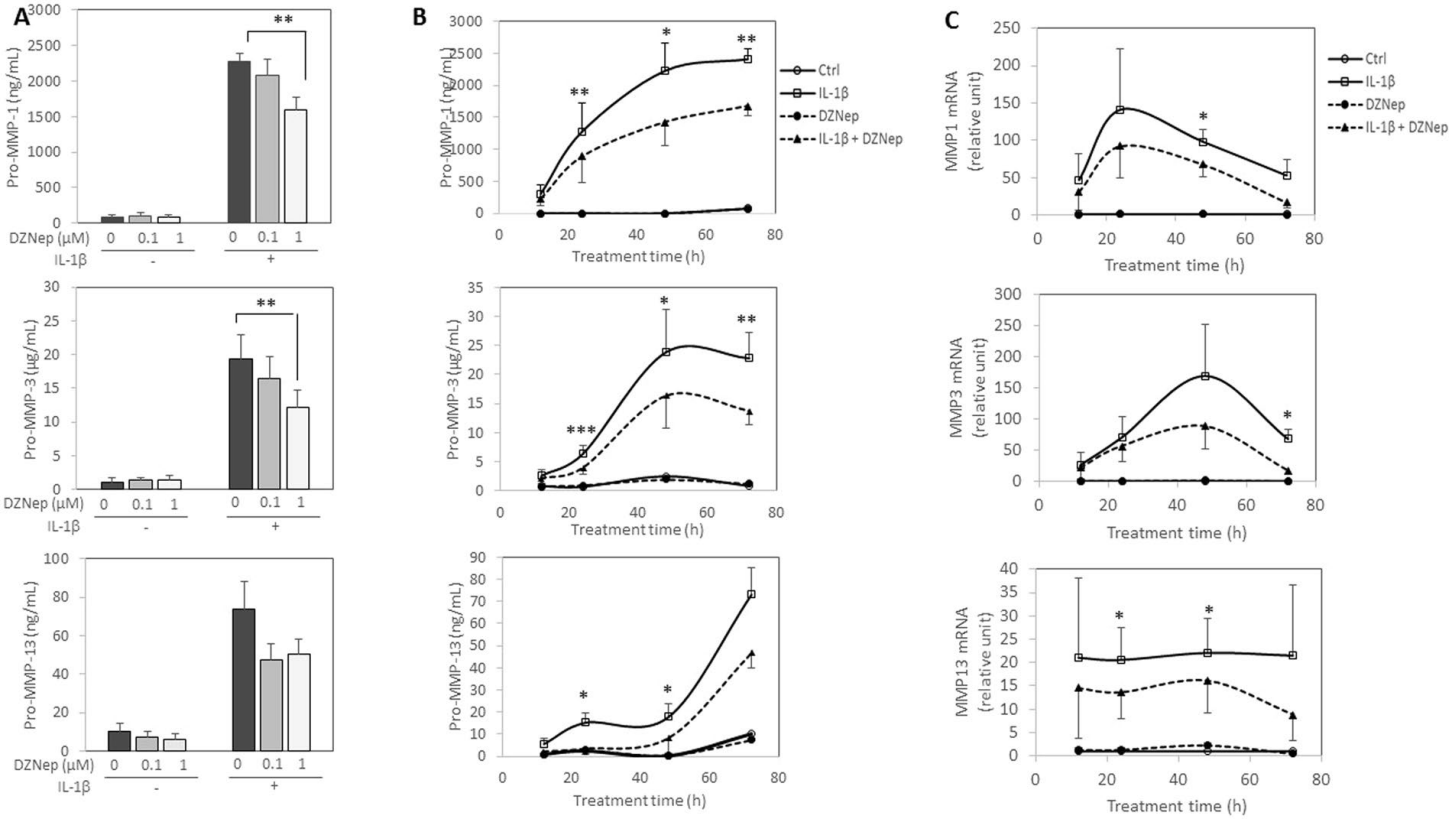
DZNep reduced MMP release and mRNA expression of MMPs induced by IL-1β. (**A**) Primary chondrocytes were treated or not with DZNep (0 µM, 0.1 µM or 1 µM) in the presence or absence of IL-1β (1 ng/mL) for 72 h. (**B**–**C**) Primary chondrocytes were treated or not with DZNep (1 µM) in the presence or absence of IL-1β (1 ng/mL) for 12 h, 24 h, 48 h or 72 h. After treatment, protein levels of MMP-1, MMP-3 and MMP-13 were determined in culture medium by ELISA assay (**B**). RNA was also extracted, and relative expression of *MMP-1*, *MMP-3* and *MMP-13* were determined in cells by real-time RT-PCR, using *GAPDH* expression as reference (**C**). Data are expressed as means ± SEM. Statistical significance between “IL-1β” and “IL-1β + DZNep” conditions are represented. *p-value ≤ 0.05; **p-value ≤ 0.01; ***p-value ≤ 0.001.

We pursued this study by analysing the effect of DZNep on IL-1β-induced expression of MMP-1, MMP-3 and MMP-13 at protein level. DZNep treatment dose-dependently reduced IL-1β-induced release of MMPs (Fig. 3A). The effects were statistically significant for MMP-1 and MMP-3 with 1 µM of DZNep. For MMP-13, we observed the same pattern, but the decrease did not reach statistical significance. Time course experiments (from 12 h to 72 h) indicated that DZNep significantly reduces MMP-1, MMP-3 and MMP-13 release from 24 h of treatment (Fig. 3B). Similar effects were observed at mRNA level after 48 h of DZNep-treatment in chondrocytes (Fig. 3C). Interestingly, DZNep was also able to counteract MMP release induced by IL-1β in synoviocytes (data not shown).

### DZNep reduces MAPK activation by IL-1β

**Figure 4:**
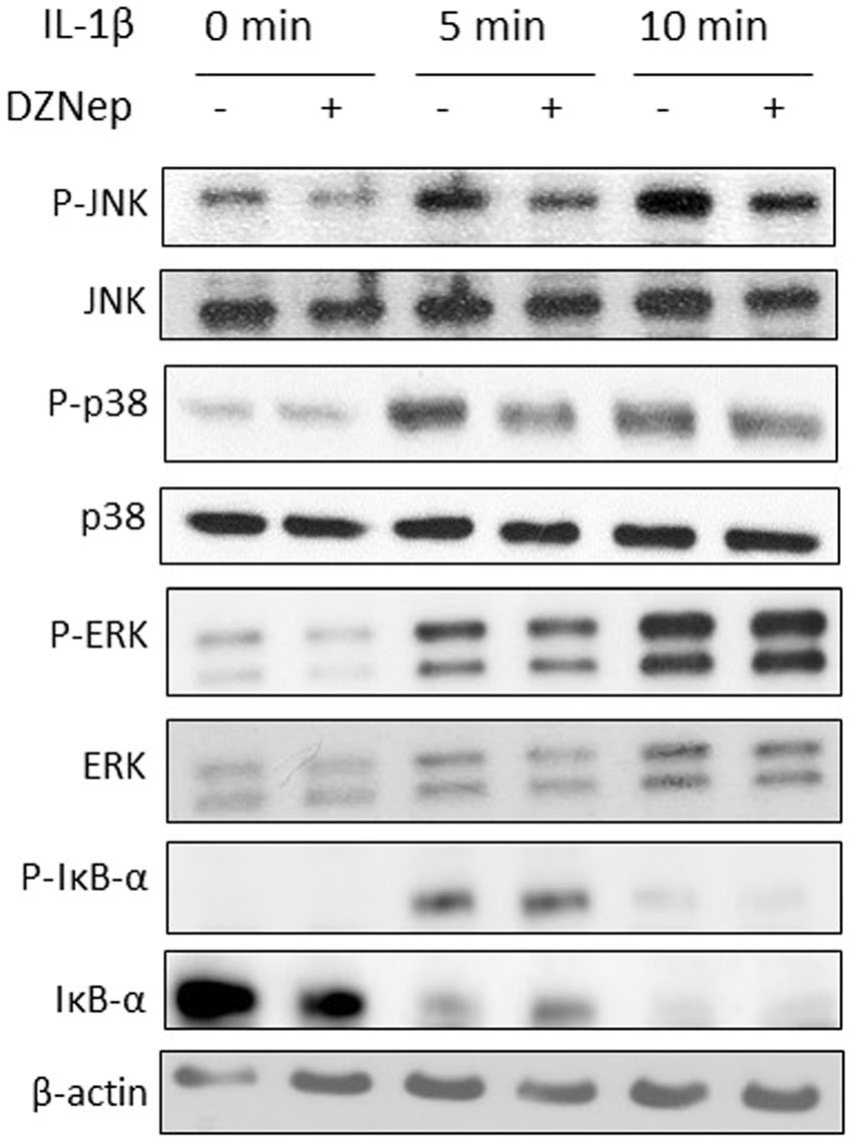
DZNep reduced MAPK pathway activation by IL-1β. Primary chondrocytes were treated or not with DZNep (1 µM) in the presence of IL-1β (1 ng/mL) for 0 min, 5 min or 10 min. After treatment, proteins were extracted, and levels of P-JNK and JNK, P-p38 and p38, P-ERK and ERK, P-IκB-α and IκB-α were determined in cells by western blotting.

Furthermore, we investigated signalling pathways induced by IL-1β, namely MAPK (p38, ERK and JNK) and NFκB pathways (Fig. 4). Western-blot analyses indicated that DZNep-treatment reduced IL-1β-induced phosphorylation of JNK and p38. Concerning NFκB pathway, we could not detect any changes in IκB-α phosphorylation or degradation induced by IL-1β in the presence of DZNep.

### DZNep counteracts the IL-1β effect on expression of genes involved in cartilage matrix degradation

To identify IL-1β-target genes that are regulated by DZNep, we compared gene expression profiling of chondrocytes treated or not by DZNep, in the presence of IL-1β. We paid attention to genes whose expression was regulated by IL-1β with a fold-change above 2 and counteracted by DZNep with a fold-change above 1.6.

**Figure 5:**
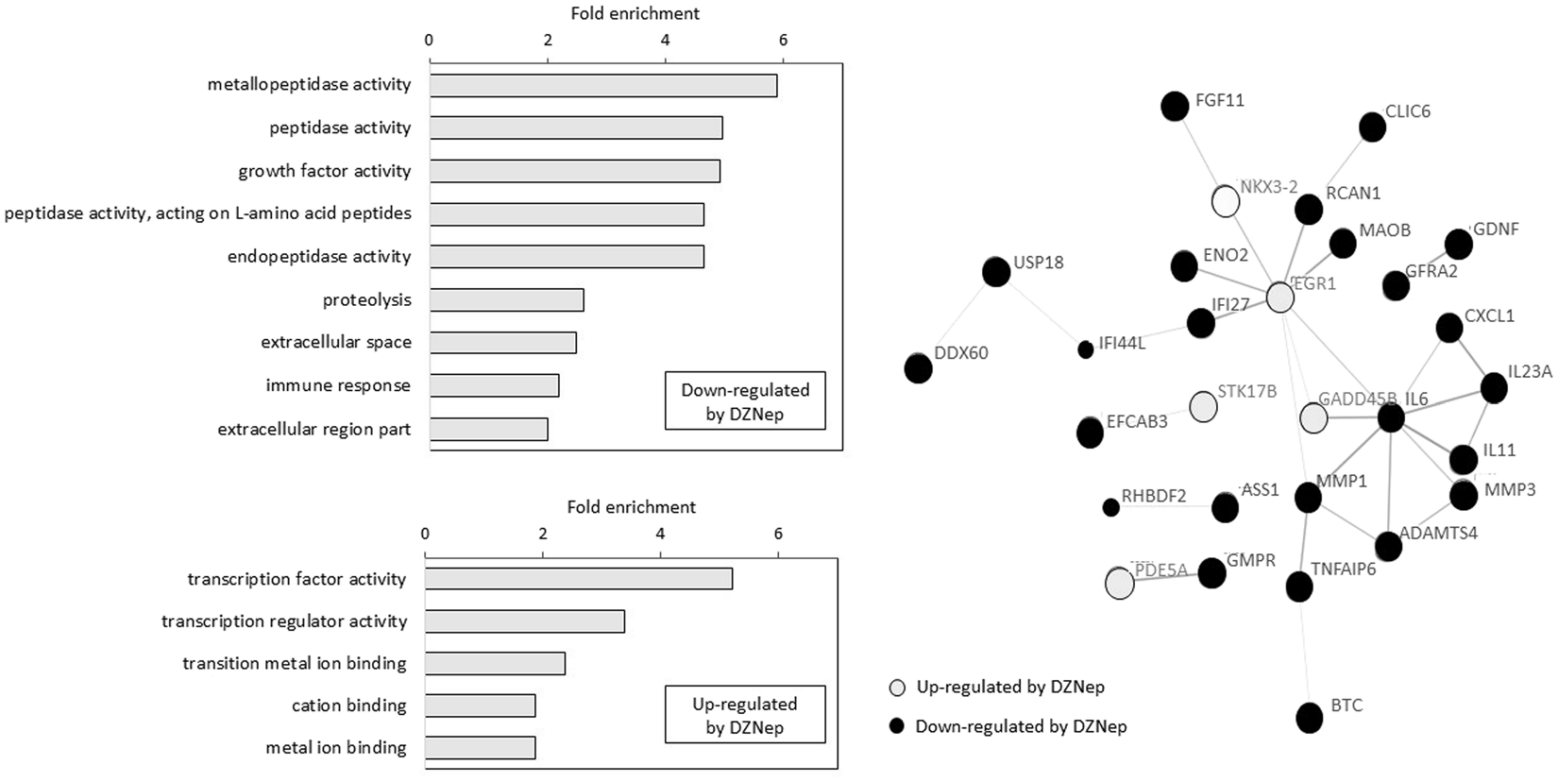
Gene ontology enrichment and PPI network of genes IL-1β-target genes regulated by DZNep. (**A**) Gene ontology enrichment of the genes regulated by DZNep in IL-1β-treated chondrocytes, using DAVID tool. (**B**) Confidence view of the PPI network built by STRING. Disconnected nodes are hidden.

Among the 936 genes up-regulated by IL-1β, 62 corresponding to 55 unique protein-coding genes were down-regulated by DZNep (Suppl. Table 1). Gene Ontology analysis (Fig. 5A, Suppl. Table 2) showed that these genes are enriched in molecular functions which can be ranged in two groups “peptidase activity/endopeptidase/metallopeptidase activity/proteolysis”, and “growth factor activity”. These genes included *MMP1*, *MMP3*, *ADAMST4*, *C1S* for the first group, and *IL-6* and *CXCL1* for the second one.

Conversely, among the 863 genes down-regulated by IL-1β, 29 entities corresponding to 26 unique protein-coding genes were up-regulated by DZNep. These genes are enriched for the molecular function “transcription factor activity/transcription regulator activity” and “transition metal ion binding/cation binding/metal ion binding” (Fig. 5A, Suppl. Table 3).

PPI network of the 81 proteins encoded by DEGs was built using STRING. This network was significantly enriched in protein-protein interactions (30 interactions, p-value = 1.78 × 10^−10^). It was centred on EGR1, which seemed to play a central role considering that it had 8 directly related proteins (Fig. 5B).

**Figure 6:**
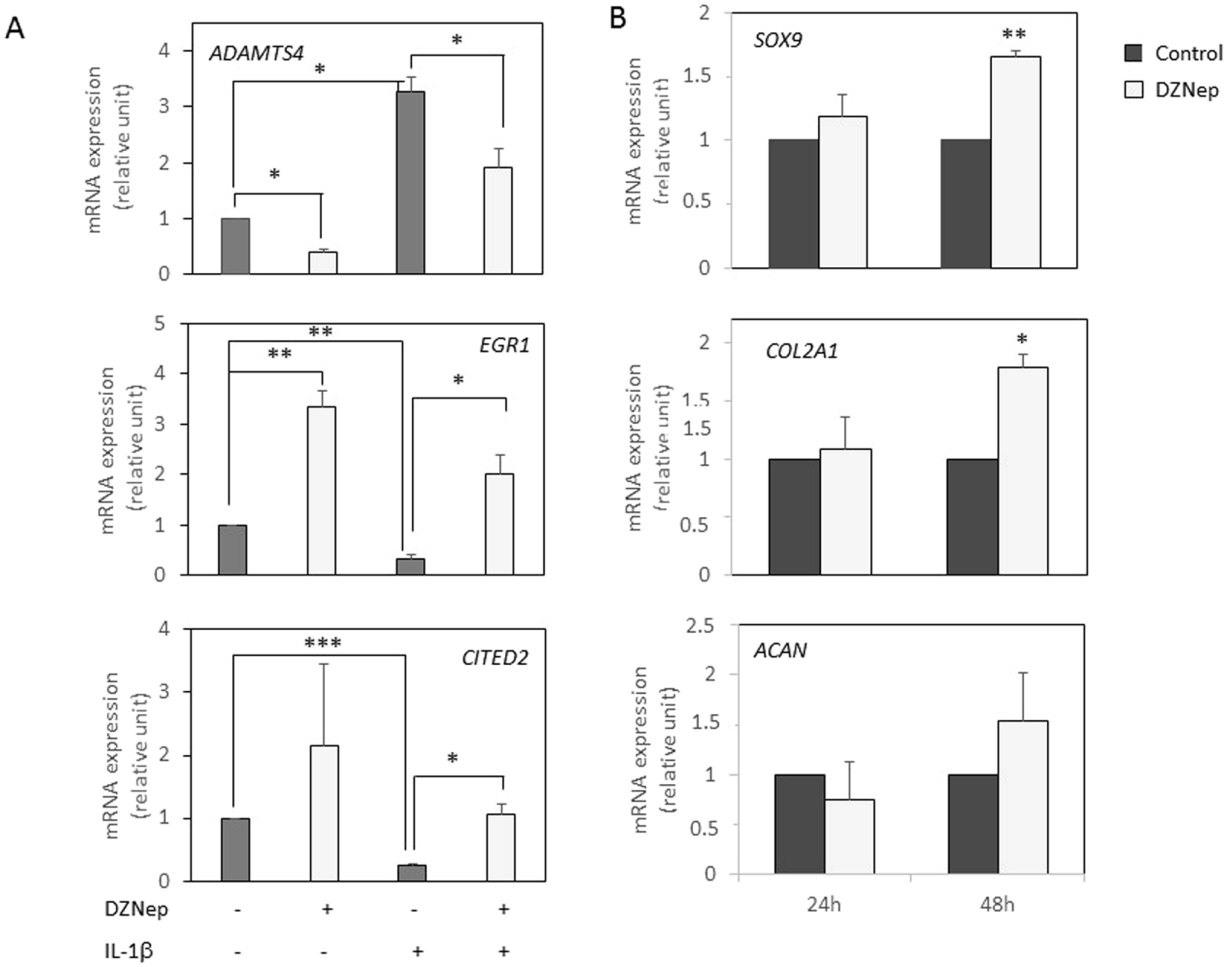
DZNep upregulated *COL2A1* and *SOX9* expression. Primary chondrocytes were treated or not with DZNep (1 µM) in the presence or absence of IL-1β (1 ng/mL) for 24 h (**A**), or in the absence of IL-1β (1 ng/mL) for 24 h or 48 h (**B**). After treatments, mRNA were extracted, and relative gene expression was determined by real-time RT-PCR, using *GAPDH* expression as reference. Data are expressed as means ± SEM. Statistical significance are represented.*p-value ≤ 0.05, **p-value ≤ 0.01., ***p-value ≤ 0.001.

Furthermore, we performed RT-PCR on several relevant genes. As expected, DZNep reduced the IL-1β-induced expression of *ADAMTS4*, and counteracted the IL-1β-induced reduction of *EGR1* but also *CITED2*, a gene known to inhibit MMP expression (Fig. 6A).

### DZNep has chondroprotective effects

Furthermore, we also paid attention to DEG when chondrocytes were treated by DZNep alone (in the absence of IL-1β). A total of 925 DEGs including 392 up- (283 unique protein-coding genes) and 533 down-regulated genes (413 unique protein-coding genes), with a fold-change above 1.6, were identified in chondrocytes treated with DZNep compared to untreated chondrocytes (Suppl. Table 4). These genes are enriched in numerous biological processes, namely “Wnt signaling pathway” (TCF7, WNT3, NR4A2, SOX4, SNAI2, FZD4, DISC1), “osteoblast differentiation” (MSX2, RSPO2, GDF10, COL6A1, RPS11, SNAI2, RBMX, MYBBP1A, IGFBP5) and “cell division” (KIFC1, NEK2, ARF6, AURKA, SPC24, FAM83D, SPC25, KIF2C, CDCA8, CDCA7, OIP5, CDCA2, BUB1, SKA1, CCNA2, CDCA5, CDK1, KIF11, SYCE3, CDC23, BIRC5, TACC3, CDC25C, UBE2C, CCNB2, MAD2L1, SPAG5, KNL1, ZWINT, CENPW, BUB1B), but also “regulation of chondrocyte differentiation” (ADAMTS7, GREM1, SNAI2). Also, there was enrichment in cellular components, such as “extracellular matrix” (RPL35A, HIST4H4, LGALS1, VIM, DCN, CFP, VWF, RPS19, RPS17, HTRA1, TGFBI, COL1A2, TGM2, COL6A1, RPS13, RPL11, RPS20, RPS11), and KEGG pathways, in particular for “mineral absorption” (MT1M, MT1A, ATP1B2, MT2A, MT1B, SLC40A1, MT1F).

These data are in agreement with literature since EZH2 has been recently reported to regulate chondrocyte proliferation and hypertrophy through Wnt signalling pathway regulation^12, 13^. Also, they suggest that DZNep might have chondroprotective effects. So, we investigated the expression of several essential genes in chondrocytes (Fig. 6B). Interestingly, *SOX9* and *COL2A1* were up-regulated by DZNep in chondrocytes. However, this effect was statistically significant only after 48 h-treatment, this could explain why these genes were not identify in the microarray experiment.

## Discussion

Osteoarthritis is characterized by irreversible degradation of articular cartilage due to the overexpression of MMPs and reduction of anabolic activity of chondrocytes, and is still difficult to treat. In the present study, we bring out the anti-inflammatory, anti-catabolic and chondroprotective effects of DZNep, a SAHH inhibitor known to inhibit EZH2 methylase.

Interestingly, we showed, in an *in vitro* model of inflammatory joint diseases, that DZNep reduces the activation of MAPK pathways (p38 and JNK), decreases the production of inflammatory molecules, such as NO and PGE2, and regulates the expression of collagenases (MMP-1, and -13), stromelysins (MMP-3) and aggrecanases (ADAMTS-4) in IL-1β-treated chondrocytes. These anti-inflammatory and anti-catabolic effects of DZNep could be mediated (at least in part) by CBP/p300-interacting transactivator with ED-rich tail 2 (CITED2, previously identified as MRG1 or p35srj). Indeed, we show that this key transcriptional modulator in inflammation and stress responses, is up-regulated by DZNep in chondrocytes both in the absence or presence of IL-1β. Interestingly, in chondrocytes, CITED2 overexpression is known to result in the reduction of MMP-1 and MMP-13 expression^14, 15^. In addition, *in vivo*, reduced CITED2 expression in cartilage contributes to OA pathogenesis, and its restoration in OA joints by gene transfer reduces cartilage degradation, and MMP-13 and ADAMTS-5 expression^16^. This suggests that DZNep may act, in chondrocytes, as an anti-inflammatory agent, which protects against the catabolism of extracellular matrix. This is in agreement with recent observations of Chen and collaborators who show that EZH2 inhibition, by EPZ005687, delays OA development in experimental osteoarthritis model in mice, and reduced the expression of the enzymes of extracellular matrix breakdown such as MMP-3, MMP-13 and ADAMTS-5 in OA cartilage^13^. In addition, these researchers show that EZH2 inhibition reduces chondrocyte hypertrophy, *in vitro* and *in vivo*, by regulating Wnt signalling pathways^13^. This is coherent with our transcriptomic analysis which shows an enrichment in cellular functions linked to “Wnt signalling pathway” and “osteoblast differentiation” in DZNep-treated chondrocytes.

Besides the reduction of cartilage extracellular matrix breakdown, DZNep could have chondroprotective effects. Indeed, we found that, in human chondrocytes, it upregulates *SOX9* and *COL2A1*, two major genes involved in cartilage anabolism. *SOX9* is a crucial transcriptional factor for chondrogenesis and chondrocyte differentiation. It is a transactivator of many genes preferentially expressed in non-hypertrophic chondrocytes, including *COL2A1*. This latter encodes type II collagen, the major hyaline cartilage matrix component.

Our whole-transcriptome data and PPI analysis revealed that *EGR1* (also named *NgfiA, Krox24, Zif268*), is upregulated by DZNep in both unstimulated and IL-1β-stimulated chondrocytes, and it may play a central role in DZNep-treated chondrocytes. Interestingly, this transcriptional factor is known to functionally interact with PRC complex and EZH2^17^, and is one of the major regulator of Sox9 expression in chondrocytes. It is able to bind to *SOX9* promoter and favors its transcription^17^.

In conclusion, DZNep has anti-inflammatory and protective effects in chondrocytes. It represses the IL-1β-induced expression of enzymes involved in cartilage breakdown, and induces expression of chondrogenic factors, such as *COL2A1* and *SOX9*. These effects seem be related to *CITED2* and *EGR1* expression. These results indicate that developing inhibitors of S-adenosylmethionine-dependent methyltransferases may be beneficial for the prevention or therapeutic treatment of osteoarthritis.

## Methods

### Reagents

Reagents were supplied by *Invitrogen* (*Fisher Bioblock Scientific*, Illkirch, France) unless otherwise noted. IL-1β (*Sigma-Aldrich*, St. Quentin Fallavier, France) was resuspended in phosphate buffered saline (PBS) with bovine serum albumin (BSA). Oligonucleotides were supplied by *Eurogentec* (Angers, France). DZNep-HCl was provided by *R&D Biosystems* (Lille, France) and resuspended in PBS.

### Cell culture and treatments

Human articular chondrocytes (HAC) were prepared from femoral head obtained after total hip replacement in osteoarthritis patients. The experimental protocol was approved by local ethical committee, named “Comité de Protection des Personnes Nord Ouest III” (agreement #A13-D46-VOL.19). Informed consent was obtained from all participants. They all signed agreement forms before the surgery, according to local legislation.

Cells were isolated and cultured as previously described^18^.

### Survival assay

Cell survival was assessed by tetrazolium colorimetric WST-1 assay (*Roche Diagnostics*, Meylan, France). Chondrocytes were plated into 96-well microtiter plates. At the end of treatment, 10 μl of WST-1 solution were added to wells. Optical density was measured on a spectrophotometer plate reader (Multiskan GO spectrophotometer, *Thermo Scientific*, France) at 450 nm. Survival was calculated according to the formula: OD_test_/OD_control_.

### S-Adenosyl-L-Homocysteine (SAH) assay

SAH accumulation was assayed using commercially available ELISA kit (*Cell Biolabs*, Inc, Lille, France). Forty µg of proteins extracted from cells using RIPA buffer were used. Absorbance was determined at 450 nm with a wavelength correction set at 540 nm (Multiskan GO spectrophotometer, *Thermo Scientific*, France).

### Nitric Oxide (NO) assay

Generation of NO was determined by measuring nitrite accumulation in culture supernatants using Griess reagent (1% sulphanilamide and 0.1% N-(1-naphthyl)-ethylenediamine dihydrochloride in 5% H3PO4, *Sigma-Aldrich*). Sample and Griess reagent were mixed and incubated for 5 min. The absorption was estimated by a plate reader (Multiskan GO spectrophotometer, *Thermo Scientific*, France) at 540 nm. Sodium nitrite (NaNO_2_, *Sigma-Aldrich*) was used as positive control.

### Metalloproteinases (MMPs) and PGE2 release assay

MMPs and PGE2 released into conditioned media was quantified using commercially available enzyme immunoassay kit (*R&D Biosystems*). Absorbance was determined at 450 nm with a wavelength correction set at 540 nm (Multiskan GO spectrophotometer, *Thermo Scientific*, France).

### RNA isolation and real-time reverse transcription-polymerase chain reaction (RT-PCR)

RNA was extracted with Trizol reagent according to the manufacturer’s condition (*Invitrogen*), and reverse transcribed into cDNA as previously described^18^. Amplification of the generated cDNA was performed by real-time PCR in Step One Plus Real Time PCR system (*Applied Biosystems*) with appropriate primers. The relative mRNA level was calculated with the 2^−ΔΔCT^ method.

### Protein extraction and western blotting

Cells were lysed and western blotting performed as previously described^19^. Following antibodies were used: Phospho-SAPK/JNK Thr183/Tyr185 (#9251), SAPK/JNK (#9252), Phospho-p38 MAPK Thr180/Tyr182 (#9211), p38 MAPK (#9212), Phospho-p44/42 MAPK (Erk1/2) Thr202/Tyr204 (#4347), p44/42 MAPK (Erk1/2) (#4695), Phospho-IkappaB-α (Ser32) (#9241) and IκB-α (#9242), from *Cell signalling*, β-actin (sc-47778), goat anti-mouse IgG-HRP and goat anti-rabbit IgG-HRP from *Santa Cruz biotechnology*.

### RNA Isolation and gene expression microarray analysis

Total RNA was isolated from cell culture using NucleoSpin RNA (*Macherey-Nagel*, Hoerdt, France), according to the manufacturer’s instructions. RNA quantity and quality were measured on a MultiSkan GO spectrophotometer with a µDrop Plate (*ThermoFisher Scientific*, France). RNA integrity was assessed on a 2100 Bioanalyzer (*Agilent Technologies France*, Les Ulis, France) using RNA 6000 Nano (*Agilent Technologies*), according to the manufacturer’s instructions. RNA integrity numbers above 8 were considered suitable for microarray analysis. Two-color microarray-based gene expression analysis was performed, according to the manufacturer’s instructions (*Agilent Technologies*). Briefly, 100 ng of total RNA were amplified and labeled using a Low input Quick Amp Labeling kit, Two-color (*Agilent Technologies*), and hybridized to a Human Gene Expression 4 × 44 K v2 Microarray (design ID 026652, *Agilent Technologies*). Slides were scanned on a G2505C Microarray Scanner (*Agilent Technologies*). Raw data were extracted and Lowess normalized using Feature Extraction software (v. 10.7.3, *Agilent Technologies*), and analyzed using GeneSpring GX software (v. 13.1.1, *Agilent Technologies*). Microarray probes with a signal that is not positive and significant or not above the background were filtered out.

### Gene–annotation enrichment analysis and Protein-Protein Interaction (PPI) network construction

Gene-annotation enrichment among the genes showing significant differential expression was performed with the Functional annotation tool of the Database for Annotation, Visualization and Integrated Discovery (v. 6.7, DAVID) tool^20^ considering the biological processes option (GOTERM_BP_FAT), the cellular compartment option (GOTERM_CC_FAT), the molecular function option (GOTERM_MF_FAT) or the Pathways database (KEGG_Pathway). Enrichments with a p-value < 0.05 were considered as significant.

Protein-protein interaction network among DEGs was constructed using the Search Tool for the Retrieval of Interacting Genes/Proteins (v. 10, STRING). Networks with a p-value ≤ 0.05 were considered significantly enriched in interactions.

### Statistical analysis

Statistical analyses were performed using R. Data were tested for normality using a Shapiro-Wilk test. The data were analysed using repeated measures Anova (Fisher test). Post-hoc analyses were done using a paired t-tests (alternative less). Differences between groups were considered statistically significant when p-value below 0.05 were measured.

### Data availability statement

The datasets generated during and/or analysed during the current study are available from the corresponding author on reasonable request.

## Electronic supplementary material

Suppl table legends

Suppl table 2

Suppl table 3
