# Supplementary material for: Anti-inflammatory and chondroprotective effects of the S-adenosylhomocysteine hydrolase inhibitor 3-Deazaneplanocin A, in human articular chondrocytes
Source: Sci Rep. 2017 Jul 25;7:6483. doi: 10.1038/s41598-017-06913-6 (PMC5526903; doi:10.1038/s41598-017-06913-6)
Supplement: Supplementary file 1 — Suppl table legends [file 41598_2017_6913_MOESM1_ESM.doc]

**Anti-inflammatory and chondroprotective effects of the S-adenosylhomocysteine hydrolase inhibitor 3-Deazaneplanocin A, in human articular chondrocytes**

**Juliette Aury-Landas^1^*, Céline Bazille^1,2^*, Lyess Allas^1^, Sara Bouhout^1^, Christophe Chesneau^3^, Sylvain Leclercq^1,4^, Karim Boumédiene^1^, Catherine Baugé^1^**^#^

**Suppl table legends**

**Suppl table 1: List of IL-1β-target genes which are regulated by DZNep in chondrocytes.**

*See excel file*

**Suppl table 2: Enrichment analysis from IL-1β-target genes which are down-regulated by DZNep in chondrocytes.** Analysis considering the biological processes option in DAVID (GOTERM_BP_FAT), the cellular compartment option (GOTERM_CC_FAT) or the molecular function option (GOTERM_MF_FAT) as indicated in the first column.

| Category | Term | Genes | Fold Enrichment | PValue |
| --- | --- | --- | --- | --- |
| GOTERM_MF_FAT | GO:0008237~metallopeptidase activity | CPD, MMP3, MMP1, ADAMTS4 | 5.89 | 0.026 |
| GOTERM_MF_FAT | GO:0008233~peptidase activity | QPCT, USP18, C1S, CPD, MMP3, MMP1, ADAMTS4, PSMB9 | 4.97 | 0.001 |
| GOTERM_MF_FAT | GO:0008083~growth factor activity | CXCL1, IL6, BTC, FGF11, GDNF, IL11 | 4.94 | 0.005 |
| GOTERM_MF_FAT | GO:0070011~peptidase activity, acting on L-amino acid peptides | USP18, C1S, CPD, MMP3, MMP1, ADAMTS4, PSMB9 | 4.66 | 0.002 |
| GOTERM_MF_FAT | GO:0004175~endopeptidase activity | C1S, MMP3, MMP1, ADAMTS4, PSMB9 | 4.66 | 0.018 |
| GOTERM_BP_FAT | GO:0006508~proteolysis | QPCT, USP18, C1S, CPD, MMP3, MMP1, ADAMTS4, PSMB9 | 2.61 | 0.025 |
| GOTERM_CC_FAT | GO:0005615~extracellular space | CXCL1, SECTM1, IL6, IL23A, SFRP1, BTC, ANGPTL1, MMP3, IL11, ADAMTS4 | 2.49 | 0.011 |
| GOTERM_BP_FAT | GO:0006955~immune response | CXCL1, SECTM1, IL6, IL23A, BST2, IFI44L, C1S, SEMA4D, GBP4, PSMB9 | 2.19 | 0.027 |
| GOTERM_CC_FAT | GO:0044421~extracellular region part | CXCL1, SECTM1, IL6, IL23A, SFRP1, BTC, ANGPTL1, MMP3, MMP1, IL11, ADAMTS4 | 2.00 | 0.031 |

**Suppl table 3: Enrichment analysis from IL-1β-target genes which are up-regulated by DZNep in chondrocytes.** Analysis considering the biological processes option in DAVID (GOTERM_BP_FAT), the cellular compartment option (GOTERM_CC_FAT) or the molecular function option (GOTERM_MF_FAT) as indicated in the first column.

| Category | Term | Genes | Fold Enrichment | PValue |
| --- | --- | --- | --- | --- |
| GOTERM_MF_FAT | GO:0003700~transcription factor activity | EGR1, NKX3-2, RUNX1T1, CITED2 | 5.21 | 0.030 |
| GOTERM_MF_FAT | GO:0030528~transcription regulator activity | EGR1, NKX3-2, RUNX1T1, TTF2, CITED2 | 3.39 | 0.041 |
| GOTERM_MF_FAT | GO:0046914~transition metal ion binding | EGR1, TP53I3, RRM2, PDE5A, RUNX1T1, RACGAP1, TTF2 | 2.37 | 0.041 |
| GOTERM_MF_FAT | GO:0043169~cation binding | EGR1, TP53I3, ATP9A, RRM2, F13A1, PDE5A, RUNX1T1, RACGAP1, TTF2 | 1.88 | 0.047 |
| GOTERM_MF_FAT | GO:0046872~metal ion binding | EGR1, TP53I3, ATP9A, RRM2, F13A1, PDE5A, RUNX1T1, RACGAP1, TTF2 | 1.88 | 0.047 |

**Suppl table 4: List of genes regulated by DZNep in human articular chondrocytes in the absence of IL-1β**

*See excel file*
